# DIA proteomics data from a UPS1-spiked *E.coli* protein mixture processed with six software tools

**DOI:** 10.1016/j.dib.2022.107829

**Published:** 2022-01-31

**Authors:** Clarisse Gotti, Florence Roux-Dalvai, Charles Joly-Beauparlant, Loïc Mangnier, Mickaël Leclercq, Arnaud Droit

**Affiliations:** a Proteomics Platform, CHU de Québec - Université Laval Research Centre, Québec City, Québec G1V 4G2, Canada; b Computational Biology Laboratory, CHU de Québec - Université Laval Research Centre, Québec City, Québec G1V 4G2, Canada

**Keywords:** Data Independent Acquisition, Complex proteomic standard, Software tools benchmark, Spiked UPS1 human proteins

## Abstract

In this article, we provide a proteomic reference dataset that has been initially generated for a benchmarking of software tools for Data-Independent Acquisition (DIA) analysis. This large dataset includes 96 DIA *.raw* files acquired from a complex proteomic standard composed of an *E.coli* protein background spiked-in with 8 different concentrations of 48 human proteins (UPS1 Sigma). These 8 samples were analyzed in triplicates on an Orbitrap mass spectrometer with 4 different DIA window schemes. We also provide the spectral libraries and *FASTA* file used for their analysis and the software outputs of the six tools used in this study: *DIA-NN, Spectronaut, ScaffoldDIA, DIA-Umpire, Skyline and OpenSWATH*. This dataset also contains post-processed quantification tables where the peptides and proteins have been validated, their intensities normalized and the missing values imputed with a noise value. All the files are available on ProteomeXchange. Altogether, these files represent the most comprehensive DIA reference dataset acquired on an Orbitrap instrument ever published. It will be a very useful resource to the proteomic scientists in order to assess the performance of DIA software tools or to test their processing pipelines, to the software developers to improve their tools or develop new ones and to the students for their training on proteomics data analysis.

## Specifications Table

**Table:** 

Subject	Biological sciences: omics: proteomics
Specific subject area	Benchmarking of DIA proteomics acquisition and processing
Type of data	MS raw Software outputs Tables
How the data were acquired	Proteomics standards composed of an *E.coli* protein background spiked-in with 48 human proteins (UPS1-Sigma) at 8 known concentrations were analyzed by Liquid Chromatography - tandem Mass Spectrometry (LC-MS/MS) on an Orbitrap Fusion mass spectrometer (Thermo Fisher Scientific). The instrument was operating in Data-Independent Acquisition with 4 different window schemes. The resulting raw files were processed with 6 software tools with or without the use of a previously acquired spectral library.
Data format	Raw (*.raw, .mzML, .mzXML*) Analyzed (software outputs) Filtered (*.blib, .tsv., .csv, .txt*)
Description of data collection	The Thermo raw files were processed with 6 DIA software tools with the use of a spectral library (Spectronaut, DIA-NN, Skyline, ScaffoldDIA, OpenSWATH) or using only a FASTA file (Spectronaut, DIA-NN, DIA-Umpire, ScaffoldDIA). The software outputs were validated at 1% FDR or *q*-value < 0.01.
Data source location	CHU de Québec - Université Laval Québec, QC, Canada
Data accessibility	All the data (raw files, converted mzML and mzXML files, spectral libraries files, software outputs and validated precursor tables) is available here: Repository name: ProteomeXchange Data identification number: PXD026600 Direct URL to data: http://proteomecentral.proteomexchange.org/cgi/GetDataset?ID=PXD026600
Related research article	*Gotti C, Roux-Dalvai F, Joly-Beauparlant C, Mangnier L, Leclercq M, Droit A. Extensive and Accurate Benchmarking of DIA Acquisition Methods and Software Tools Using a Complex Proteomic Standard. J Proteome Res. 2021 Sep 2.* doi: 10.1021/acs.jproteome.1c00490. *PMID:**34472865**.*

## Value of the Data

• This dataset is the most comprehensive DIA dataset acquired on an Orbitrap mass spectrometer with a complex proteomic standard.
• In comparison to other proteomic reference dataset, it contains spiked-in proteins at known concentrations to assess the ability of proteomic pipelines to recover low abundance proteins.
• Proteomic scientists could use it to better understand the performance of DIA software tools and choose the best pipeline for their study.
• Students could use it for their training on DIA proteomics data analysis.
• Software developers could use it to assess and improve their tools for the detection of low abundance proteins.
• This dataset can be considered as a reference for the development of new DIA analysis tools.

## 1 Data Description

**Fig. 1:**
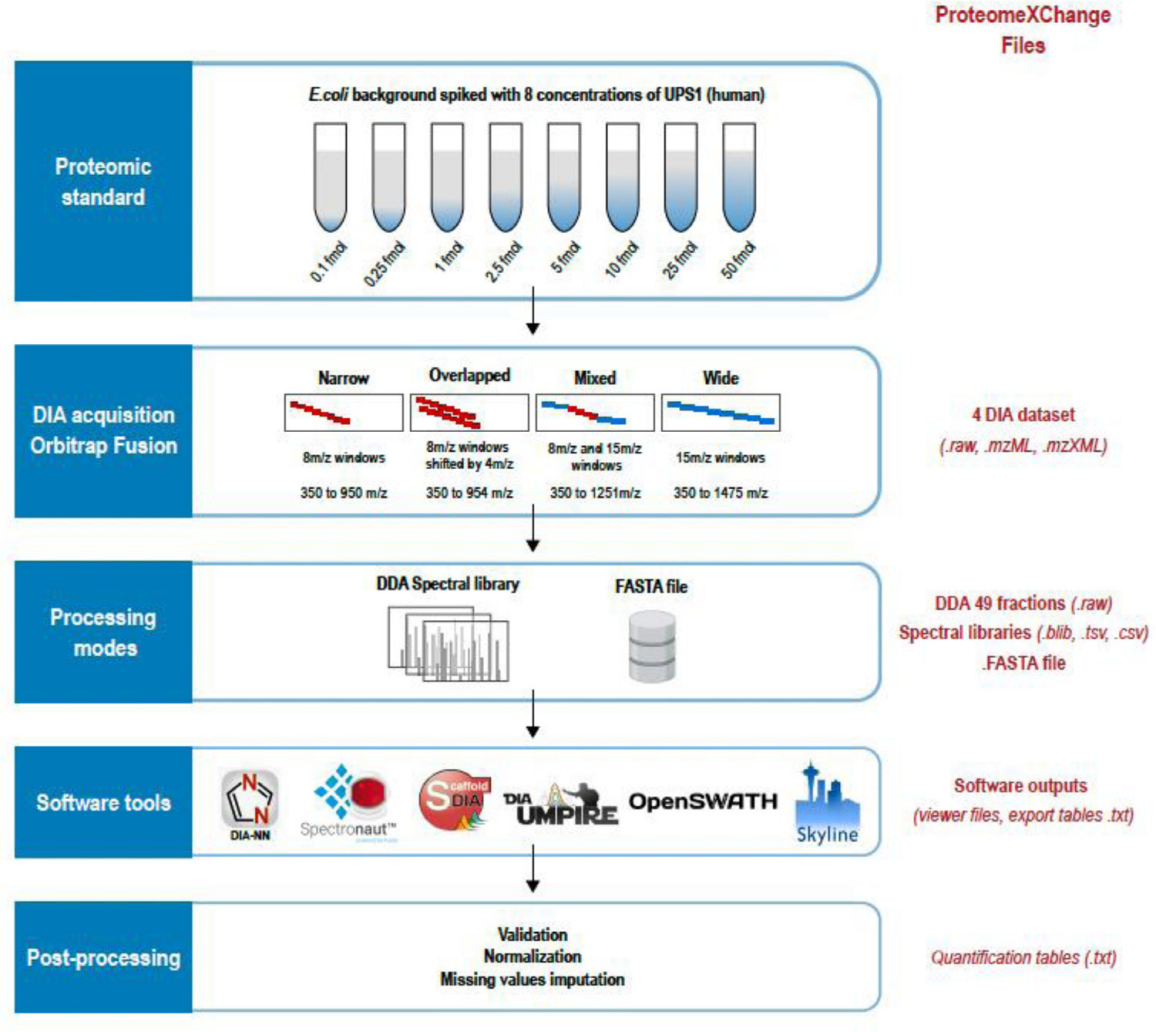
**Analysis workflow.** The proteomic standard composed of 8 E.coli protein samples spiked with Sigma-UPS1 proteins was analyzed with 4 different acquisition modes, 2 processing modes and 6 DIA software tools. The final data was validated, normalized and its missing values imputed with a noise value. At each step, the corresponding files are provided in ProteomeXchange PXD026600 (in red on the left side).

The dataset provided in this article has been initially generated with the aim to benchmark DIA acquisition methods and software tools [1]. As shown on Fig. 1, we used a complex proteomic standard composed of an *E.coli* protein background spiked-in with 8 different concentrations of the 48 UPS1 human proteins (Sigma) ranging from 0.1 to 50 fmol per microgram of *E.coli* proteins. These samples were analyzed on an Orbitrap mass spectrometer operating in Data-Independent Acquisition mode. Four different DIA acquisition schemes were used since narrow windows are expected to provide less complex DIA spectra (less precursors are selected for fragmentation) but wide windows can better cover the mass range in an appropriate chromatographic cycle time. Two other schemes using overlapped windows or mixed window sizes were also tested (Table 1).

**Table 1:** **Acquisition parameters in DIA mode on the Orbitrap Fusion mass spectrometer.** Four acquisition methods were used all with the same MS full scan while the DIA MS/MS was performed with various window sizes and mass ranges.

		Narrow	Wide	Overlapped	Mixed
MS	Scan range	350-1800			
	Orbitrap resolution	60,000			
	AGC target	4.00E+05			
	Max Injection time	50 ms			

MS/MS	Scan range (m/z)	350-950	350-1475	350-954	455-1251
	DIA window width	8 m/z	15 m/z	two cycles of 8m/z windows shifted by 4 m/z	8 m/z in 455-711 range, 15 m/z for the 350-455 and 711-1251 ranges
	Collision energy	HCD 35%			
	Orbitrap resolution	15,000			
	AGC target	5.00E+04			
	Max Injection time	22 ms			

For each DIA scheme *(Narrow, Wide, Mixed* and *Overlapped*), three injections (analytical replicates) of the 8 samples were done. Therefore we provide 4 datasets of 24 raw files in Thermo .*raw* file format and converted .*mzML* or .*mzXML* formats.

Six public or proprietary software tools were used for the processing of the raw files (Table 2). Some of them required the use of a DDA (Data Dependent Acquisition) spectral library (*Library* mode) (Skyline and OpenSWATH), one only required a FASTA file (*FASTA* mode) (DIA-Umpire) and others can do both (Spectronaut, DIA-NN, ScaffoldDIA). For the *Library* processing mode, we used (and provide here) DDA *.raw* files acquired on the same instrument from 48 peptide fractions of an *E.coli* protein extract and 1 unfractionated sample containing a protein digest of *E.coli* background spiked-in with UPS1 proteins. Two spectral libraries were generated using these 49 DDA *.raw* files and are provided as well as *.blib, .tsv* and *.csv* files. The *.FASTA* file (containing *E.coli* proteome and UPS1 proteins sequences) used for the *FASTA* mode is given as well. Finally, the 96 DIA .*raw* files were processed with the 6 software tools with the use of these libraries or with the *FASTA* file. The corresponding software outputs are provided as viewer files that can be re-open in the corresponding software tool and as untreated *.txt* export tables.

**Table 2:** **Processing of DIA files.** The table shows the software tools used for DIA files processing along with their version, raw files conversion, use of FASTA and/or Library mode, search parameters and data filtering settings.

	Version	Raw file conversion	FASTA mode	Library mode	Search Parameters	Data filtering
DIA-NN [4]	02/04/2020	.mzML	yes (DIA spectral library from DIA files)	yes (Skyline .blib exported to .tsv)	Maximum Missed Cleavages: 2; Fixed Modifications: carbamidamethyl (C); Variable modifications Oxidation (M); Min. Fragments: 4; Max. Fragments: 6; Peptide charge: 2+ to 4+; Other settings (see Gotti *et al.*Supp Table S1 [1])	1% FDR or q-value < 0.01 at precursor, peptide and/or protein level (see Gotti et al.Supp Table S1 [1])
DIA-Umpire [5]	2.0	.mzXML	yes (DIA spectral library from DIA files)	no		
OpenSWATH (diaproteomics) [6,7]	1.1.0	.mzML	no	yes (Skyline .blib exported to .tsv)		
ScaffoldDIA (Proteome Software, Inc.) [8]	2.1.0	none	yes (in silico Prosit library)	yes (Skyline .blib)		
Skyline [2]	20.2.0.343	.mzML	no	yes (Skyline .blib)		
Spectronaut (Biognosys AG) [3]	14.10.20122	none	yes ('directDIA' mode)	yes (Spectronaut library)		

We finally provide *.txt* precursor quantification tables in which the data is validated for protein and peptide identification, normalized and the missing values imputed with a noise value as described in the Table 3.

**Table 3:** **Post-processing of precursor tables.** For each software tool, the information on outliers’ removal, normalization and missing value imputation is given along with the criteria to consider a precursor as identified and quantifiable. The steps were performed in R in the same order than shown in the table from to top to bottom.

	DIA-NN [4]	DIA-Umpire [5]	OpenSWATH (diaproteomics) [6,7]	ScaffoldDIA (Proteome Software, Inc.) [8]	Skyline [2]	Spectronaut (Biognosys AG) [3]
Precursor tables filtering	decoy and contaminant features removal					

Outliers removal	no	no	yes, cut-off at 1	no	no	yes, cut-off at 10

Normalization	automatic; use normalized precursor intensity	manual; median normalization of precursor intensity using a fcator calculated on all precursors of each injection	manual; median normalization of precursor intensity using a fcator calculated on all precursors of each injection	automatic; use normalized precursor intensity	manual; median normalization of precursor intensity using a fcator calculated on all precursors of each injection	automatic; use normalized precursor intensity

E.coli precursor identified	If at least one intensity value for the 24 injections of the same dataset was reported					

UPS1 precursor identified	If at least one intensity value among the 3 analytical replicates of each sample was reported					

Precursor (E.coli or UPS1) quantifiable	If 3 intensity values were reported in the 3 analytical replicates of a sample in at least one sample of the 8 UPS1 concentrations.					

Missing value imputation	For the quantifiable precursors, missing values were imputated by a noise value corresponding to the first percentile of all precursor intensities for each sample injection					

Quantification value	Precursor intensities were summed by stripped sequences to obtain peptide quantification and by accession number for protein quantification					

**Fig. 2:**
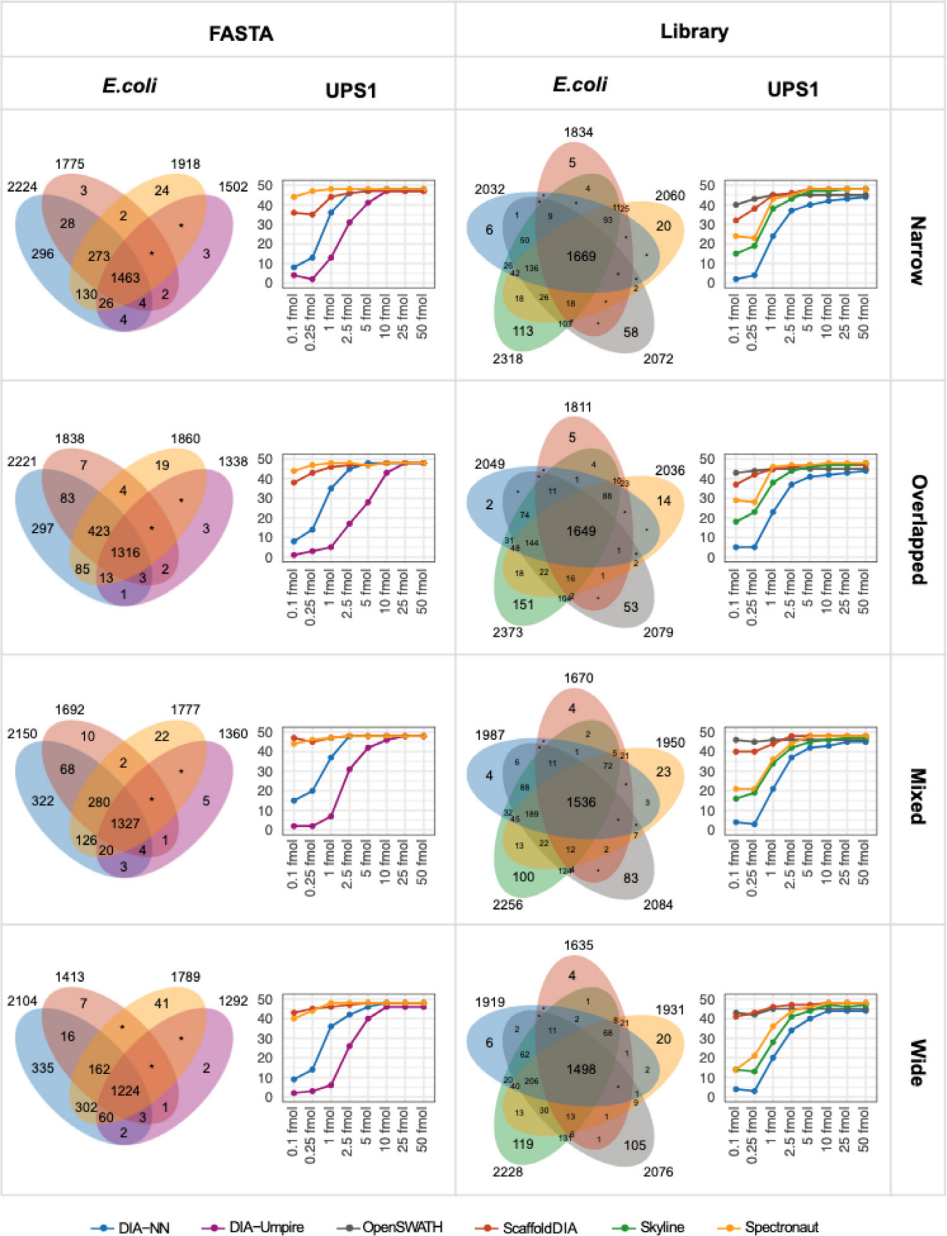
*E.coli* and UPS1 protein identifications for each analysis pipeline. The Venn diagrams on the left side show the overlap of E.coli protein identifications between the different software used (an E.coli protein is considered as identified if it has been found in at least 1 of 24 files of one dataset). The dot plots on the right side show the number of UPS1 proteins identified for each of the 8 samples (an UPS1 protein is considered as identified if it has been found in at least 1 of the 3 analytical replicates). The same graphs are presented for each DIA acquisition scheme (Narrow, Overlapped, Mixed and Wide) and each processing mode (FASTA or Library). Software tools: DIA-NN (blue), Spectronaut (yellow), ScaffoldDIA (orange), DIA-Umpire (purple), Skyline (green), OpenSWATH (grey).

Fig. 2 and Supplementary Table 1 give the number of proteins identified in each pipeline. The Venn diagrams (Fig. 2, left panels) show the number of *E.coli* protein identifications and their overlap between the different software tools used. For each of them we identified between 1292 and 2373 *E.coli* proteins. On the right panels, we can observe how many of the 48 UPS1 proteins were identified in each sample. This number is decreasing with the concentration of UPS1 spiked in the *E.coli* background.

## 2 Experimental Design, Materials and Methods

### 2.1 Preparation of the proteomic standard

*E.coli* protein extract was obtained from a broth culture *Escherichia coli* (strain #CCRI-12923, CCRI, Québec, Canada) in Brain Heart Infusion (BHI) medium at 8 × 10^8^ cfu/mL. The culture was centrifuged at 10,000 x g for 15 min and stored at -20°C. Proteins were extracted by resuspension of the pellet in the extraction buffer (50 mM ammonium bicarbonate, 1% sodium deoxycholate and 20 mM 1,4 dithiothreitol), heated 10 min at 95°C and sonicated 15 minutes with 30s/30s ON/OFF cycles at high intensity (Bioruptor, Diagenode). The lysed cells were then centrifugated at 13,000 x g for 10 min to remove debris and the protein concentration in the supernatant was determined by Bradford Assay. The concentration was then adjusted at 0.1µg/µL in extraction buffer.

A vial of Universal Proteomic Standard-1 (UPS1, Sigma) containing 48 human proteins (5pmol each) was serially diluted using the *E.coli* protein extract to obtain 8 concentrations of UPS1 per microgram of E.coli (50, 25, 10, 5, 2.5, 1, 0.25 and 0.1 fmol/µg). Reduction and alkylation of cysteines was performed by heating the sample for 30 min at 37°C followed by addition of 50mM iodoacetamide and incubation for 30 min. The pH was then adjusted to 8.0, trypsin enzyme (Promega) was added at a ratio of 1:50 (enzyme:protein) and the samples were incubated at 37°C. The reaction was stopped by acidification to pH2.0 with formic acid. The samples were then centrifugated at 16,000 x g for 5 minutes. The peptides contained in the supernatants were purified on Oasis HLB cartridge 10 mg (Waters) and vacuum dried.

### 2.2 Mass spectrometry

The samples were resuspended in 2% acetonitrile, 0.05% TFA and for each one, an equivalent of 1µg peptides was analyzed by LC-MS/MS an U3000 NanoRSLC liquid chromatography system (ThermoScientific, Dionex Softron GmbH, Germering, Germany) in line with an Orbitrap Fusion Tribrid – ETD mass spectrometer (ThermoScientific, San Jose, CA, USA). Peptides were concentrated at 20µL/min (loading solvent: 2% acetonitrile/0.05% trifluoroacetic acid) on a 300 mm i.d x 5 mm, C_18_ PepMap100, 5 mm, 100 Å precolumn cartridge (Thermo Fisher Scientific) for 5 minutes. Then, the separation was performed on a PepMap100 RSLC, C_18_ 3 mm, 100 Å, 75 µm i.d. x 50 cm length column (Thermo Fisher Scientific) using a 90 min linear gradient from 5-40% solvent B (A: 0.1% formic acid, B: 80% acetonitrile/0.1% formic acid) at 0.3 µL/min.

The mass spectrometer was operated in Date Independent Acquisition (DIA) mode. Four methods (*Narrow, Wide, Overlapped, Mixed*) with different DIA window schemes were used as described in Table 1.

### 2.3 Generation of spectral libraries

250µg of *E.coli* protein extract was prepared as described above and digested in the same conditions was high-pH fractionated on a Agilent Extend C_18_ (1.0 mm x 150 mm, 3.5 µm) column using an Agilent 1200 Series HPLC system. Peptides were separated at 1 mL/min by a gradient of 5–35% solvent B for 60 minutes and 35–70% solvent B for 24 minutes (A: 10 mM ammonium bicarbonate, pH 10; B: 90% acetonitrile/10% ammonium bicarbonate pH 10). 48 fractions were collected. A sample of 200 fmol UPS1 per µg of *E.coli* extract was also prepared as described for the proteomic standard to complete the spectral library with UPS1 human proteins.

These 49 samples were analyzed on the same instrument and with the same chromatographic conditions than for the proteomic standard but the mass spectrometer was operated in Data Dependent Acquisition (DDA) mode with the following settings:

- MS: Full scan MS 350-1800 m/z; Orbitrap resolution: 120,000; AGC target 4e5; Max injection time 50 ms
- MS/MS: To speed mode 3s; Isolation window: 1.6 m/z; HCD fragmentation with 35% collision energy; Orbitrap resolution: 15,000; AGC target 5e4; Max injection time 22 ms; dynamic exclusion: 30s.

Database searching was performed in Proteome Discoverer 2.3.0.523 using the Mascot search engine version 2.5.1 (Matrix Science) with the following settings:

*Protein Database:*

• *E.coli* database (UniProt Reference Proteome – Taxonomy 83333 – Proteome ID UP000000625 – 4312 entries – 2016.03.15)
• UPS1 database (downloaded from Sigma – 48 entries)

*Enzyme Name:* trypsin

*Maximum Missed Cleavage Sites:* 2

*Precursor Mass Tolerance:* 10ppm

*Fragment Mass Tolerance:* 25 mmu

*Dynamic Modifications:* Oxidation (M)

*Static Modifications:* Carbamidomethyl (C)

Peptide identifications were then filtered at 1% False Discovery Rate (FDR) using Percolator (v 2.0).

Mascot .dat files were then used to generate a spectral library in Skyline software [2] (version 20.2.0.343) through a *.blib* file. Another spectral library was generated in Spectronaut v14.10.20122 (Biognosys AG) [3] using the. pdresults file of Proteome Discoverer. Detailed parameters used to generate both spectral libraries are listed in the supplementary table S1 of the Gotti *et al.* article [1].

### 2.4 DIA file processing

The DIA files were then processed with 6 different software tools in *FASTA* mode using a single *E.coli* and UPS1 FASTA file (UniProt Reference Proteome – Taxonomy 83333 – Proteome ID UP000000625 – 4312 entries – 2016.03.15 and the 48 sequences of UPS1) or in *Library* mode with one of the Skyline or Spectronaut spectral libraries as described in Table 2. The tools were used as recommended by the user manual or by the software developers and detailed parameter settings can be found in the supplementary table S1 of the Gotti *et al.* article [1].

### 2.5 Data post-processing

All data post-processing was performed using R software [9] from precursor tables exported from each software tool. Table 3 shows the treatment applied to the data of each tool as well as the criteria to consider a precursor identified and quantifiable.

## Ethics Statement

This work does not involve human subjects, animal experiments or data collected from social media platforms.

## CRediT authorship contribution statement

**Clarisse Gotti:** Conceptualization, Investigation, Visualization. **Florence Roux-Dalvai:** Conceptualization, Investigation, Writing – original draft, Project administration. **Charles Joly-Beauparlant:** Software. **Loïc Mangnier:** Software. **Mickaël Leclercq:** Software. **Arnaud Droit:** Writing – review & editing, Supervision.

## Declaration of Competing Interest

The authors declare that they have no known competing financial interests or personal relationships that could have appeared to influence the work reported in this paper.

## Appendix Supplementary Materials

Image, application 1
